# Supplementary figures and images for: Identification of the Keratin-Associated Protein 22-2 Gene in the Capra hircus and Association of Its Variation with Cashmere Traits
Source: Animals (Basel). 2023 Sep 4;13(17):2806. doi: 10.3390/ani13172806 (PMC10487131; doi:10.3390/ani13172806)

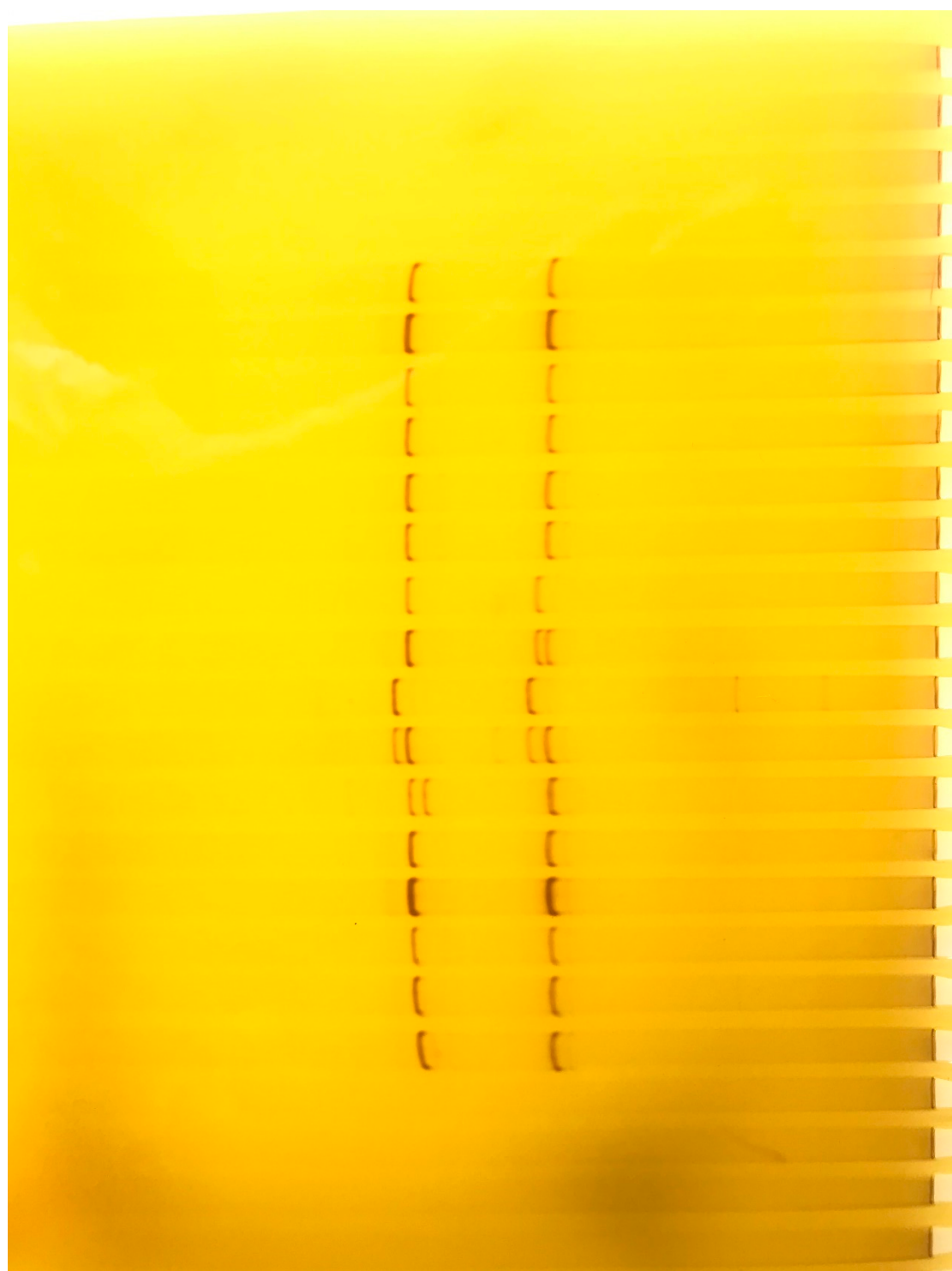

Supplement: Supplementary file 1 [file animals-13-02806-s001.zip › animals-2543791-supplementary.pdf]
